# Supplementary material for: From Micro to Macro: Avian Chromosome Evolution is Dominated by Natural Selection
Source: bioRxiv. 2024 Dec 4:2024.11.29.626112. Preprint. [Version 1] doi: 10.1101/2024.11.29.626112 (PMC11642735; doi:10.1101/2024.11.29.626112)

bioRxiv preprint doi: https://doi.org/10.1101/2024.11.29.626112; this version posted December 4, 2024. The copyright holder for this preprint (which was not certified by peer review) is the author/funder, who has granted bioRxiv a license to display the preprint in perpetuity. It is made available under aCC-BY 4.0 International license.

Supplemental Figure 1. Chromosome fission and fusion rates for 12 bird orders. Bars represent the 95% credible interval.

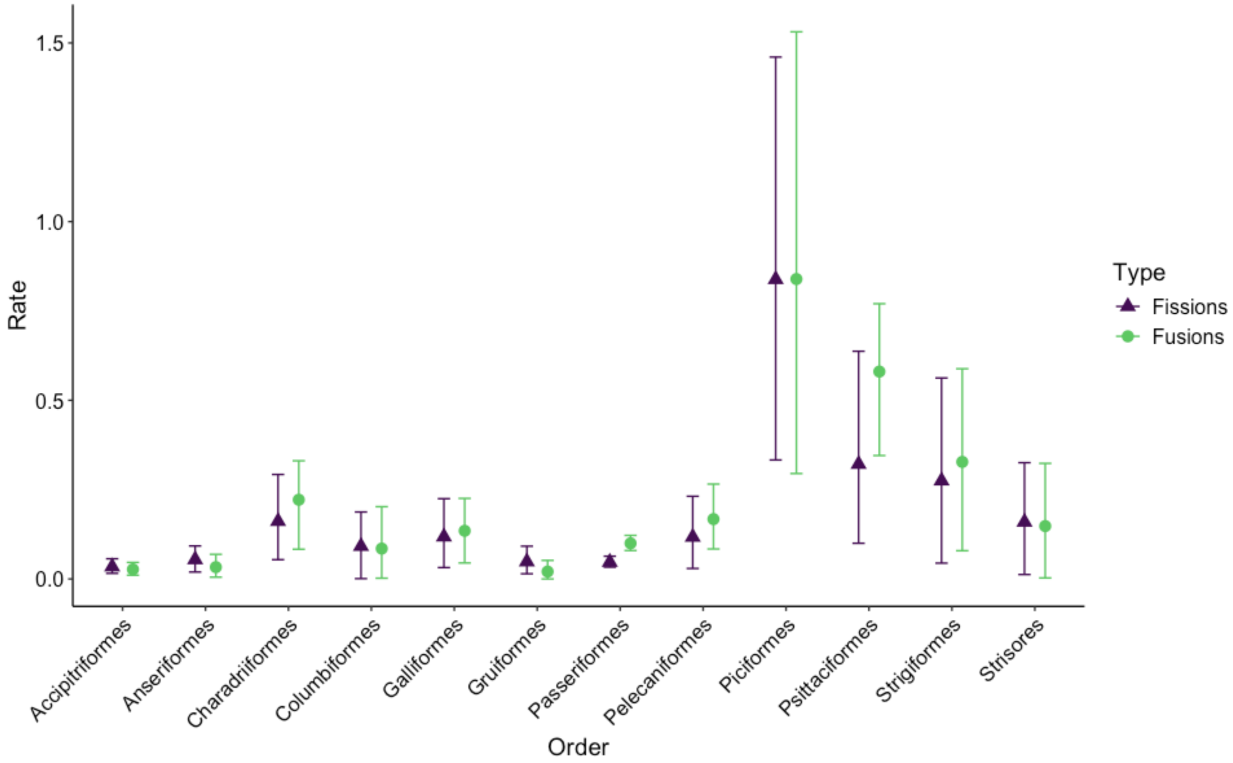

bioRxiv preprint doi: https://doi.org/10.1101/2024.11.29.626112; this version posted December 4, 2024. The copyright holder for this preprint (which was not certified by peer review) is the author/funder, who has granted bioRxiv a license to display the preprint in perpetuity. It is made available under aCC-BY 4.0 International license.

Supplemental Figure 2. Chromosome evolution in small population size and large population size lineages excluding high tip rate taxa. Each curve shows the distribution of the rate difference in small population size and large population size lineages. The bars below the curve indicate the 95% credible interval for the rate differences.

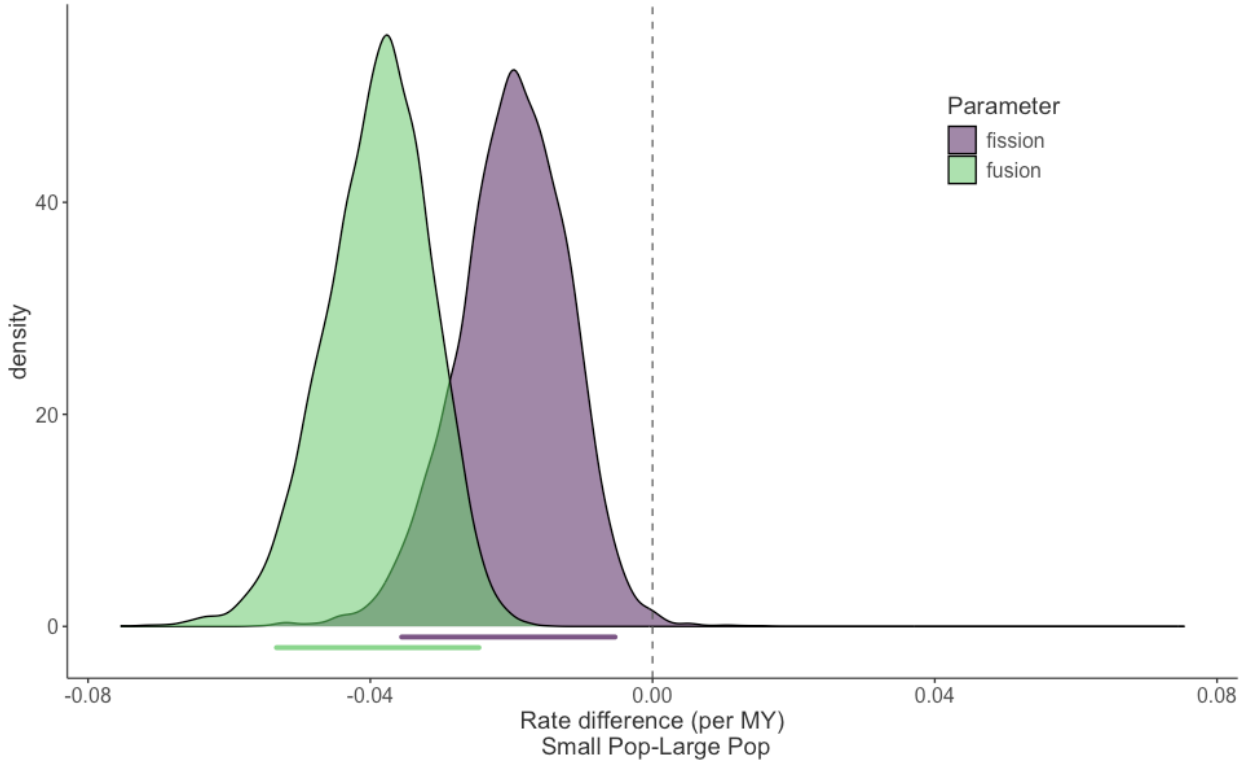

bioRxiv preprint doi: https://doi.org/10.1101/2024.11.29.626112; this version posted December 4, 2024. The copyright holder for this preprint (which was not certified by peer review) is the author/funder, who has granted bioRxiv a license to display the preprint in perpetuity. It is made available under aCC-BY 4.0 International license.

Supplemental Figure 3. Chromosome evolution in migratory and sedentary Passeriformes. Each curve shows the distribution of the rate difference in sedentary and migratory lineages within Passeriformes. The bars below the curve indicate the 95% credible interval for the rate differences.

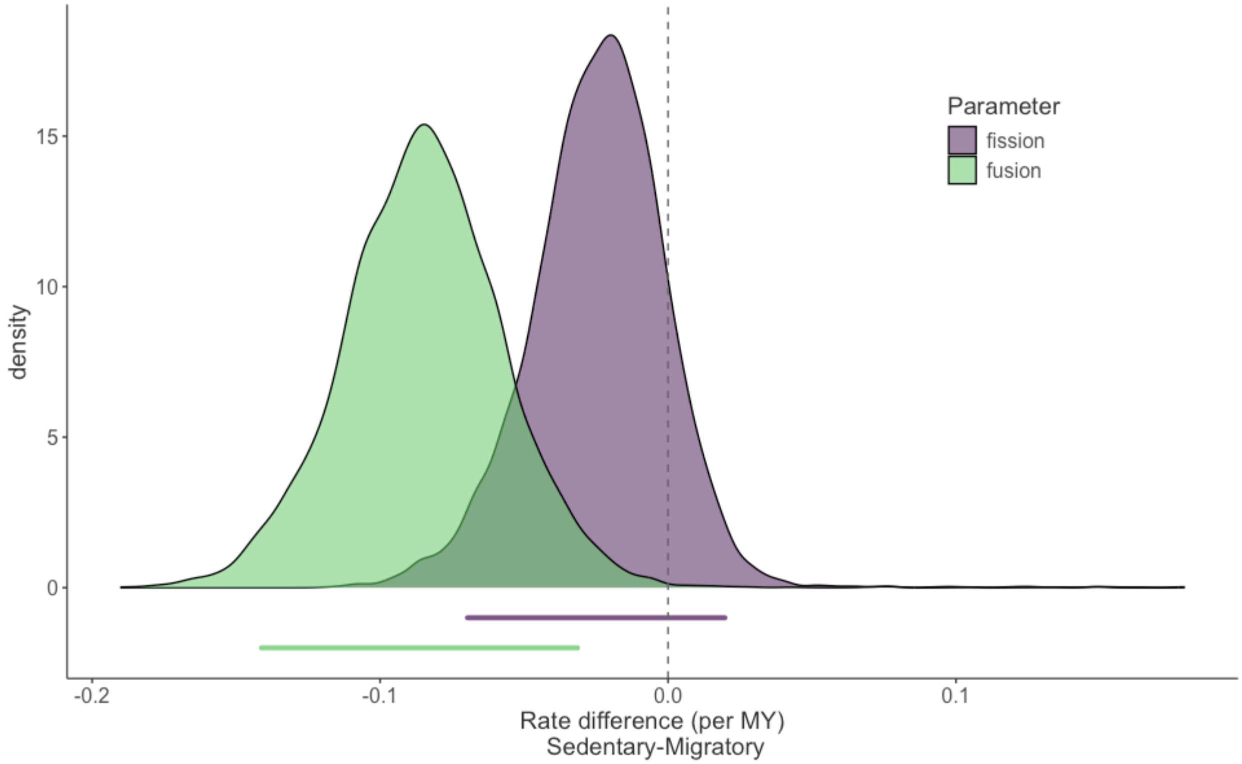

Supplement: 1 [file NIHPP2024.11.29.626112V1-supplement-1.pdf]
